# Expanded CD1c^+^CD163^+^ DC3 Population in Synovial Tissues Is Associated with Disease Progression of Osteoarthritis

**DOI:** 10.1155/2022/9634073

**Published:** 2022-08-01

**Authors:** Guowei Qiu, Sheng Zhong, Jun Xie, Hui Feng, Songtao Sun, Chenxin Gao, Xirui Xu, Bingxin Kang, Hui Xu, Chi Zhao, Lei Ran, A. Xinyu, Bo Xu, Xiaohui Meng, Lu Meng, Xiaoming Zhang, Lianbo Xiao

**Affiliations:** ^1^Shanghai University of Traditional Chinese Medicine, Shanghai, China; ^2^Department of Orthopedics, Guanghua Hospital Affiliated to Shanghai University of Traditional Chinese Medicine, Shanghai, China; ^3^Arthritis Institute of Integrated Traditional Chinese and Western Medicine, Shanghai Academy of Traditional Chinese Medicine, Shanghai University of Traditional Chinese Medicine, Shanghai 200052, China; ^4^Department of Rehabilitation, The First Affiliated Hospital of Henan University of Chinese Medicine, Zhengzhou, China; ^5^Henan University of Traditional Chinese Medicine, Zhengzhou, China; ^6^The Center for Microbes, Development and Health, Key Laboratory of Molecular Virology & Immunology, Institut Pasteur of Shanghai, Chinese Academy of Sciences/University of Chinese Academy of Sciences, Shanghai, China

## Abstract

The mechanisms underlying osteoarthritis (OA) have recently been hypothesized to involve a dysfunctional immune system. In this study, we collected synovium, synovial fluid (SF), and peripheral blood from 21 patients. Mononuclear cells were characterized using FCM. H&E staining and mIHC histological assessment of synovium were performed. Cytokine levels in the SF were measured using ELISA. We observed similar frequencies of immune cells in the synovium and SF, which were enriched in DCs. Notably, CD1c^+^CD163^+^ DC3s were expanded in the synovium and SF. Furthermore, we found that DC3s were primarily located within the ectopic lymphoid-like structure (ELLS) in close proximity to CD8^+^ T cells. Finally, the level of TNF-*α* and IL12p70 in the SF correlated with the severity of OA. These data suggest that OA is an immune system-related disease and that DC3s may play an active role in OA progression by promoting ELLS formation and inflammatory responses.

## 1. Introduction

Osteoarthritis (OA) is the most prevalent arthritic condition in the elderly (over 60 years worldwide) and thus has a high impact on patient activity and is associated with heavy economic burden [1]. Pain is the most common sign of disease and the leading cause of disability [2]. Although OA is mainly considered a degradative condition of the articular cartilage, there is increasing evidence demonstrating that OA is a low-grade inflammatory disease that affects all tissues of the joint, characterized by profound changes in intracellular mechanisms and decreased efficiency of the immune system with ageing [3].

After T cells, macrophages, and other immune cells infiltrate into joint tissues, cytokines and chemokines such as TNF-*α* and IFN-*γ* are secreted, the complement system is activated, and cartilage-degrading factors such as matrix metalloproteinases are released, causing damage to the articular cartilage [4]. There has been considerable success in the treatment of rheumatoid arthritis using anticytokine therapies [5]. However, these therapies did not show much effect in OA, highlighting the more complex nature of OA pathogenesis. Thus, a better understanding of the pathogenic mechanisms of chronic immune activation and the development of novel therapeutic strategies for OA are urgently required.

Dendritic cells (DCs) are a class of bone marrow-derived cells arising from lymphomyeloid hematopoiesis. DCs form an essential interface between the innate sensing of pathogens and the activation of adaptive immunity [6]. The initiation and control of immune responses depend on three major subsets: plasmacytoid DCs (pDCs), myeloid/conventional DC1 (cDC1s), and DC2 (cDC2s). Previously, we demonstrated that both macrophages and DCs were enriched in the joint synovium, suggesting that the type, density, and location of immune cells within the local milieu may strongly influence OA pathogenesis. Here, we aimed to evaluate and explore the full spectrum of immune cell types and DC subsets using high-dimensional flow cytometry and multiplex immunohistochemistry (mIHC). Specifically, in addition to pDCs and cDC1s, according to BTLA and CD163 expression, we further defined BTLA^+^CD163^−^ cDC2s and BTLA^−^CD163^+^ DC3s, with the latter primarily induced by inflammation [7, 8]. Several studies reported that CD163 expression associated with OA progression and pain. CD163 expression in the synovium is elevated in patients with OA compared to healthy controls [9]. The soluble form of CD163, sCD163, is cleaved from CD163 during inflammation by ADAM-17, and elevated sCD163 levels in blood reflect inflammation and radiographic severity in OA [10]. CD163 expression is associated with higher resting pain scores [10]. In the current study, significantly increased levels of inflammatory DC3s were observed in the synovium compared with PBMCs and synovial fluid (SF). In addition, DC3s and CD8^+^ T cells colocalized within ectopic lymphoid-like structures (ELLS), suggesting a role for DC3s in organizing ELLS by attracting and activating CD8^+^ T cells. Moreover, the number of ELLS is proportional to disease severity. Together, this study revealed that DC3s were highly enriched in the joints of OA patients, with potentially pathogenic roles in disease progression.

## 2. Materials and Methods

### 2.1. Study Population

**Figure 1:**
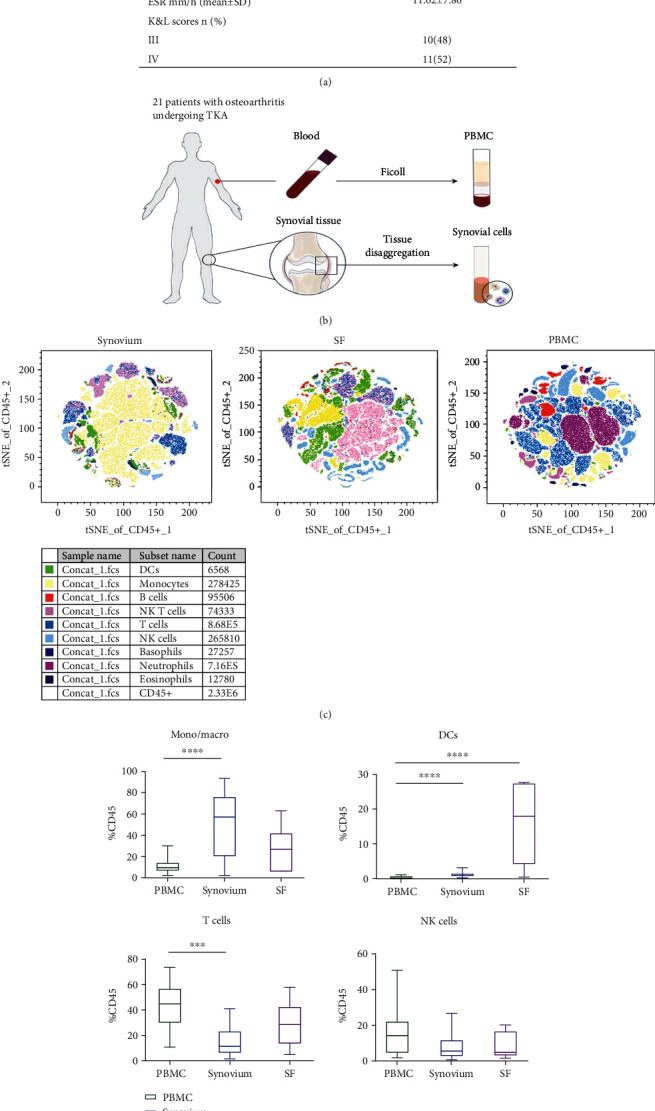
Landscape of immune cells in the synovium, SF, and paired PBMC. (a) 21 OA patients undergoing TKA were studied. Range of WBC: 3.5–9.5 × 10^−9^/L; range of CRP: ≤10 mg/L; range of ESR: 0–20 mm/h. (b) PBMC cells and tissue MNs were isolated separately from 21 OA donors. (c) Identification of 9 major immune cell subsets by tSNE of flow cytometry data of the synovium, SF, and PBMC. (d) Proportions of monocytes/macrophages, DCs, T cells, and NK cells to CD45^+^ cells in PBMC, synovium, and SF. Significance was assessed by Wilcoxon matched-pairs signed rank test. ^∗∗∗^*P* < 0.001. ^∗∗∗∗^*P* < 0.0001.

From March 2021 to June 2021, patients with OA in the Orthopedic Surgery Department of Guanghua Hospital, Shanghai University of Traditional Chinese Medicine (21 patients), were enrolled. The diagnostic criteria for OA conformed to those of the American College of Rheumatology. We used the Kellgren and Lawrence (K&L) score to assess disease severity in patients with OA through imaging. Blood testing of each patient revealed that there was no inflammatory reaction in the body before the operation. The clinical characteristics of the study population are shown in Figure 1(a). This study was approved by the Ethics Committee of Guanghua Hospital, and all study participants provided written informed consent.

### 2.2. Sample Preparation for Flow Cytometry Analysis

Before surgery, 5 mL of venous whole blood from was collected OA patients, placed in a sodium heparin anticoagulation tube, and transported at room temperature. During total knee arthroplasty (TKA) surgery, synovial tissue was collected, and the joint fluid was placed in EDTA tube and transported on ice at 4°C. Peripheral blood mononuclear cells (PBMCs) were separated by density centrifugation on Lymphoprep (Axis-Shield, Norway). For the flow cytometry assay, 100 *μ*L fluorescently labeled antibody mixture was added to each sample (containing CD16-BV510, CD33-BV711, CD56-PE, CD19-BV650, CD14-A700, CD15/CD66b-PerCP-Cy5.5, CD1c-BB515, CD45-APC-Cy7, CD3-PE-Cy5.5, BTLA-PE-Cy5, CD88-PC594, CD141-APC, FceRI*α*-Biotin/Streptavidin-BUV395, CD123-BV786, CD163-BV605, and HLADR-BV421). Meanwhile, mononuclear cells (MNs) were obtained from tissues as follows: after rinsing tissue with 1× PBS three times, weighing, and first cutting into 1 cm × 1 cm pieces with ophthalmological scissors for the mIHC assay, the remaining tissue was cut into small pieces. SF were frozen in aliquots of 100 *μ*L at -80°C for subsequent cytokine detection; digestive enzymes (type II collagenase 10 mg/mL+DNase I 100 U/mL) were then separately added to synovial tissue and SF, digested for 1 h in a 37°C shaker, and filtered through a 70 *μ*m mesh. 5 mL of MACS buffer containing 1% FBS was then added, and the sample was centrifuged at 500 × *g* for 10 min. The supernatant was discarded, 100 *μ*L of MACS buffer was added to resuspend the cells, the cells were transferred into a sterile EP tube, Zombie Yellow-BV570 was added for staining (RT, 30 min), and 100 *μ*L fluorescently labeled antibody mixture was added for cell surface staining (room temperature, 15 min). The cells were washed with 1 mL of MACS buffer (800 × *g*, 2 min), and the supernatant was discarded. Cells were then fixed in fixation buffer (eBioscience, Intracellular Fixation Buffer) for 10 min at 4°C, washed with 1 mL MACS buffer, and resuspended in 500 *μ*L MACS buffer. A BD Fortessa flow analyzer and FlowJo software (V10.7.2) were used for data analysis. The cluster diagram is completed by the plugin t-SNE in FlowJo 10.7.2 software.

### 2.3. Histological Assessment of the Synovium Samples

Synovial tissue samples were retrieved for histological analysis after paraffin embedding. Following hematoxylin and eosin (H&E) staining of 4 *μ*m thick paraffin-embedded sections, multiplex immunohistochemistry (mIHC) was performed according to the manufacturer's instructions (Thermo Fisher Scientific Logo, Opal® Kit). This process was performed using the following antibodies and fluorescent dyes in the following order: CD1c/Opal570, CD8/Opal520, and CD163/Opal650. Detailed procedures for mIHC and quantitative analysis were performed as previously reported [11]. Slides were scanned and imaged using the PerkinElmer Vectra3® platform and analyzed in batches using PerkinElmer inform and R script for the quantification of positively stained cells.

### 2.4. Cytokine Detection

The joint fluid was thawed at room temperature for 10 min. Before use, the joint fluid was centrifuged at 10,000 × *g* for 10 min and the supernatant was collected. ELISA for human TNF-*α*, IL12p70, or IL23 were performed according to the manufacturer's instructions (Absin Bioscience Inc., abs510012/abs510006/abs510013). The corresponding cytokine concentrations in each sample were obtained by referring to the standard curve.

### 2.5. Preparation and Culture of DC3s and Macrophages with T Cells

After fluorescently labeled antibody stain, DC3s (CD88^−^CD1c^+^CD163^+^) and macrophages (CD88^+^CD14^+^) were isolated from OA patients PBMCs using the BD FACSAria II system. CD3^+^ T cells were isolated from PBMCs from deidentified healthy volunteers which were isolated by Lymphoprep (density gradient separation; StemCell) and stored in liquid nitrogen until further use. For *in vitro* priming, CD3^+^ T cells were combined with DC3s or macrophages at a 10 : 1 responder-to-stimulator ratio (T cells : DC3s or macrophages) and were conducted in 96-well (1 × 10^4^ CD3^+^ T cells) plates. For cytokine analysis, cells were incubated for five days. After incubation, cells were stained with CD8, followed by intracellular staining with the following antibodies: IFNgamma, TNFalpha, and IL17 (BD Pharmingen). Cytokine-producing cells were gated according to lymphocyte FSC/SSC profile and CD8^+^ cells. Samples were fixed in 1% paraformaldehyde and stored at 4°C after staining. All flow cytometry data were collected and analyzed as detailed above.

### 2.6. Statistical Analysis

Continuous variables were compared using the *t*-test or nonparametric Mann–Whitney *U* test, as appropriate. The Kruskal-Wallis test was used for the analysis of immune cell frequencies between the study groups. In the latter case, variations in statistical significance were further subjected to post hoc pairwise analyses. The Mann–Whitney *U* test was performed to assess differences in cytokine expression in SF samples. All the reported *P* values were two-tailed. A *P* value < 0.05 was considered to show a statistically significant difference. Statistical analyses were performed using GraphPad Prism 8.3.0 software and SPSS 25.0. The number of the nearest neighbors was calculated on the assigned coordinates of each cell using R software with the spatstat package.

## 3. Results

### 3.1. Differences in Immune Cell Patterns between Synovium Tissue, SF, and PBMCs in OA Patients

OA patients undergoing total knee arthroplasty (TKA) were enrolled in our study. Classification of 10 KL3 and 11 KL4 OA patients was performed on the basis of clinical signs. Of the 21 patients identified, the mean age was 71 years, with no symptoms of inflammation (Figure 1(a)). A total of 17/21 patients with OA were female, consistent with the fact that autoimmune disease patients are most commonly female [12]. For each patient, mononuclear cells from the tissues (MNs) and peripheral blood (PBMCs) were isolated, as shown in Figure 1(b).

To characterize the immune status of the synovium, we used 18-marker high-dimensional flow cytometry to study the major immune cell populations compared to paired PBMCs and SFs. Nine immune cell subsets were annotated: neutrophils (SSC-H^hi^CD15^+^CD16^+^), eosinophils (SSC-H^hi^CD15^+^), basophils (FceRI*α*^+^HLADR^−^), T cells (CD3^+^), B cells (CD19^+^), NK cells (CD3^−^CD56^+^), NKT cells (CD3^+^CD56^+^), monocytes (CD88^+^CD14^+^), and DCs (CD123^+^HLADR^+^ pDCs, CD1c^+^ mDCs, and CD141^+^ mDCs). SFigure 1 shows the conventional manual gating strategy used to define these populations.

A comparative analysis was performed on the synovium, SF, and PBMCs. The pattern of immune cells was observed in the synovium and SF, which was completely different from that observed in PBMCs (Figures 1(c)–1(e)). Although the number of each immune cell population varied greatly among patients, the frequency of macrophages showed major differences in the synovium (50.77% ± 29.65% of CD45^+^ cells; *P* = 0.0001) compared to PBMCs (10.96% ± 6.38) (Figure 1(d), SFigure 2). The frequencies of total DCs in the SF and synovium were also significantly higher than those in the PBMCs, whereas fewer neutrophils and B cells were found. NK cells and NK T cells did not exhibit any major differences. However, a decreased percentage of T cells was observed in both the synovium and SF (~25% CD45^+^ cells). Taken together, these data showed that macrophages, T cells, and NK cells were enriched, whereas granulocytes were decreased in the synovium compared with PBMCs.

### 3.2. High Infiltration of CD1c^+^CD163^+^ DC3s in the Synovium

**Figure 2:**
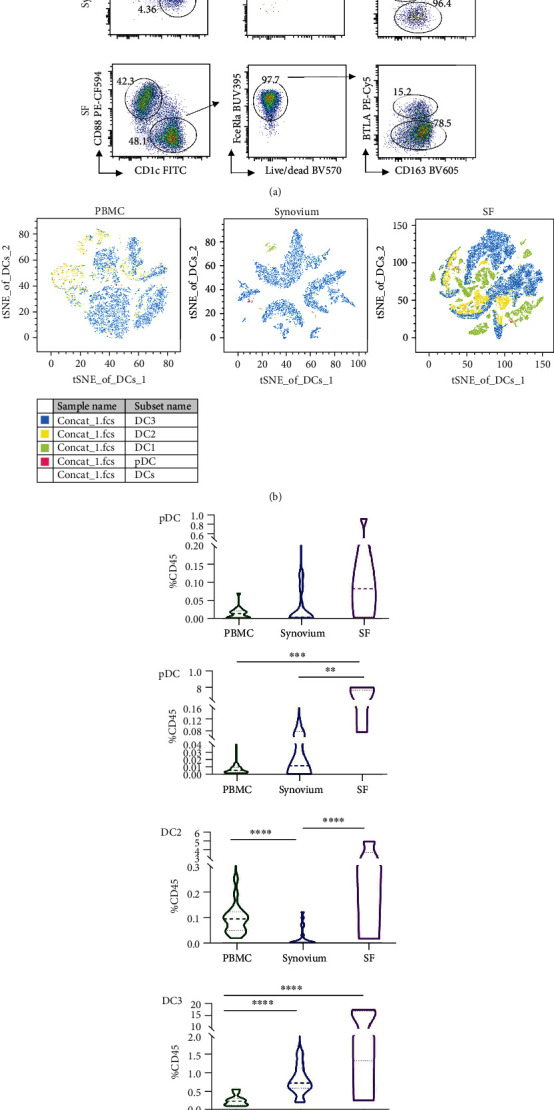
DC3s significantly infiltrated in the synovium. (a) Gating strategy for identification of DC2s and DC3s in flow cytometry. (b) 4 DC subsets were defined in PBMC, synovium, and SF (*n* = 6). (c) Histograms showing the proportion of DCs of CD45^+^ cells in the synovium (*n* = 21) compared with PBMC (*n* = 21) or SF (*n* = 6). Significance was assessed by Wilcoxon matched-pairs signed rank test. ^∗∗^*P* < 0.01. ^∗∗∗^*P* < 0.001. ^∗∗∗∗^*P* < 0.0001.

DCs comprise various subsets and exhibit distinct functions in autoimmune diseases [13]. The DC subgroups pDCs (CD123^+^), DC1s (CD123^−^CD141^+^), DC2s (CD1c^+^BTLA^+^CD163^−^), and DC3s (CD1c^+^BTLA^−^CD163^+^) were further investigated in patients with OA. Each DC subgroup was gated as shown in Figure 2(a). In line with earlier reports, the percentage of DC1s or total DCs was significantly higher in the synovium and SF than in the PBMC [14] group, whereas the percentage of pDCs did not differ between the three groups (Figure 2(b), Figure 2(c)). A similar proportion of DC2s was observed in SF and PBMCs. Intriguingly, DCs in the synovium were composed of almost DC3s. Among CD45^+^ immune cells, ~0.5% of DC3s were enriched in the synovium, while DC3 levels were found to be 10-fold higher in the SF. These results indicate that an increased number of cDCs accumulate during OA progression.

### 3.3. DC3s Organize into an Ectopic Lymphoid-Like Structure (ELLS) Associated with Disease Severity

**Figure 3:**
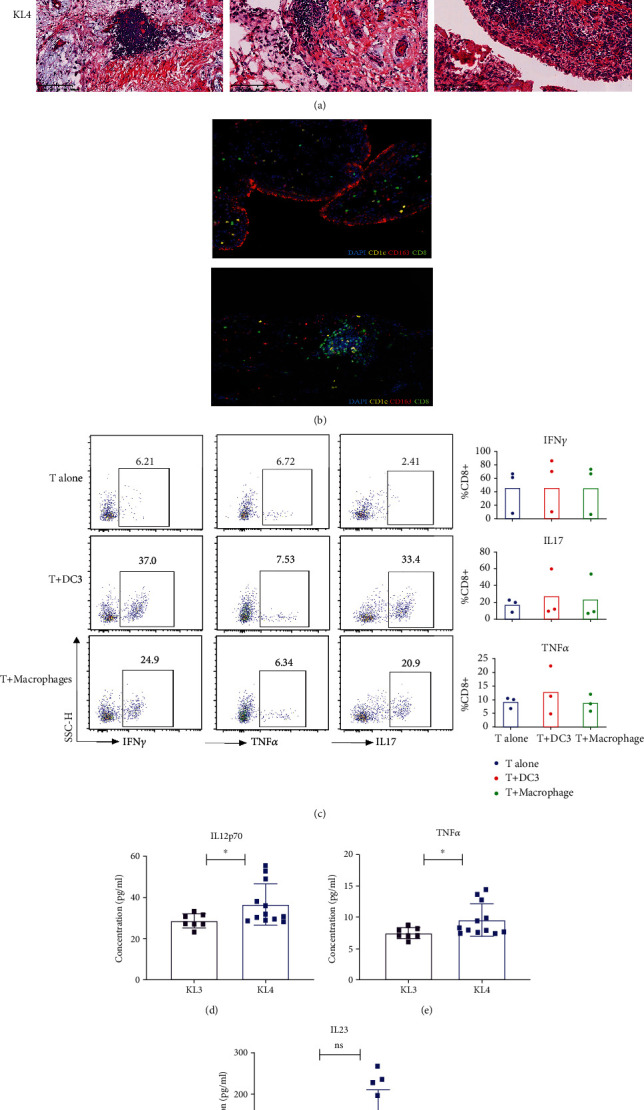
DC3s infiltrated in the synovium is correlated with CD8^+^ T cells. (a) H&E stain. (b) Representative mIHC image shows the staining for CD1c (yellow), CD163 (red), and CD8 (green) in the OA synovium. In the left picture, white arrow indicates DC3s (CD1c^+^CD163^+^). A typical ELLS consist plenty of DC3s and CD8^+^ T cells showed in the right image. Magnification, ×200. (c) Assessment of proinflammatory cytokine profiling of CD8^+^ T cells (*n* = 3). Representative flow cytometry plots (left) and accumulated data (right) to show the proinflammatory cytokines IFN-*γ*, TNF-*α*, and IL-17 secreting profile of CD8^+^ T cells following the stimulation of PMA, ionomycin, and BFA for 5 h (*n* = 3). Histograms showing higher level of (d) IL12p70 and (e) TNF-*α* in severe OA patients. (f) No significant differences of IL23 were observed. Significance was assessed by Wilcoxon matched-pairs signed rank test. ^∗^*P* < 0.05. ^∗∗^*P* < 0.01.

To better explore the extent of synovitis and the contribution of cells to the degree of inflammation, we performed histopathological analysis of synovial tissue samples collected from KL3 and KL4 OA patients. As shown in the left H&E image in Figure 3(a), the lining cells formed a single layer, and the synovial stroma showed normal cellularity with no inflammatory infiltrates. However, the presence of synovial lesions consistent with low-grade synovitis demonstrated an increase in the thickness of the lining layer and stromal cellularity, with the presence of a few, mostly perivascular lymphocytes. Comparatively, high-grade synovitis (Figure 3(a), right picture) was distinguished by the presence of a greatly thickened lining, increased infiltration of numerous lymphocytes, and typical ELLS.

Next, we applied mIHC to further investigate cellular infiltration in the OA synovium by simultaneously gating for CD1c, CD8, and CD163. CD1c^+^CD163^+^ DC3s and CD8^+^ T cells were readily detected (Figure 3(b)). Growing evidences suggest a role of CD8^+^ T cells in progression of osteoarthritis [15–17]. Activated peripheral blood CD8^+^ T cells gathered in the knee joint would cause local inflammation resulting in joint failure [18]. Recently, DC3s was proved *in vivo* to efficiently induce differentiation of CD8^+^ T cells, while DC3 infiltration was found to correlate with CD8^+^ T cell accumulation in breast tumors [7]. Consistent with this, we observed that DC3s and CD8^+^ T cells were highly enriched in ELLS. We hypothesized that ELLS might be organized by DC3s, together with CD8^+^ T cells. To confirm this, DC3s were isolated from 3 OA synovial tissues separately. We also isolated synovial macrophages aimed to understand the immunological function of DC3s and to compare it. As shown in Figures 3(c) and 3(d), the number of IFN-*γ*- and TNF-producing CD8^+^ T was increased after DC3 priming comparable to the macrophage-treated group. The involvement of IL-17-producing CD8^+^ T cells (Tc17) was also found. This is in line with the involvement of Tc17 in various conditions such as autoimmune inflammation [19, 20]. Additionally, we applied cellular phenotyping of the fluorescence image and depicted the spatial location of DC3s and CD8^+^ T cells *in situ*. We divided the DC3-CD8^+^ T cell distance as <20 *μ*m and >20 *μ*m [11] (SFigure 3A). The relative number of DC3s was calculated, and significantly higher numbers of DC3s were recovered within 20 *μ*m than beyond (SFigure 3B). In particular, ELLS were only found in <20 *μ*m positive samples including OA18, OA38, and OA39. These data suggest that the aggregation of DC3s was positively related to the formation of the ELLS structure.

DC3s are an important source of proinflammatory cytokines, including TNF-*α*, L12p70, and IL23 [7]. As shown in Figures 3(d)–3(f), the levels of TNF-*α* and IL12p70 in the SF of KL4 patients were significantly higher than those in KL3. Together, these results suggest that the increased infiltration of DC3s in joint infiltration, together with the elevated proinflammatory cytokines in SF, may play a critical role in the progression of OA.

## 4. Discussion

OA was initially defined as a disease induced by mechanical stress in the form of cartilage destruction, with minimal, if any, involvement of immune responses. Thus, OA, in contrast to rheumatoid arthritis (RA), has long been regarded as a noninflammatory disease [21–23]. However, recent studies have demonstrated that, at least in certain patients, OA is an inflammatory disease, with patients frequently found to exhibit inflammatory infiltration of synovial membranes [24, 25]. Recent studies have shown that the number of inflammatory cells in synovial tissue is higher than that in peripheral blood [26, 27]. Both macrophages and T cells play important roles in OA pathogenesis [28–31]. Consistent with previous studies, we also found higher percentages of macrophages in synovial tissue and SF than in PBMCs, whereas a decreased number of T cells was measured in synovial tissue and SF [24, 32].

Surprisingly, among CD45^+^ cells, DCs were redundant in synovial tissue and SF. DCs have the broadest range of antigen presentation. The activation of innate immunity plays a critical role in the development and progression of OA. Innate immunity, including inflammasome activation, is triggered by small endogenous molecules called damage-associated molecular patterns, which are released in the extracellular media after cell stress or damage, bind to pathogen-recognition receptors, including distinct Toll-like receptors, and activate the secretion of proinflammatory cytokines, resulting in joint inflammation. Moreover, the functional role of Toll-like receptors expressed on DCs in OA development has also been reported [33].

DCs are necessary for the activation of naïve T cells. In this study, we found that DC3s, defined as CD1c^+^CD163^+^ cells, were significantly enriched in the synovium and could prime naïve CD8^+^ T cells. In synovial tissue, CD8^+^ T cells are often located at the periphery of the aggregation [32]. As shown by our mIHC results, a substantial number of DC3s and CD8^+^ T cells were observed within the synovium. Consistent with this, higher levels of TNF-*α* and IL12p70, which are mainly secreted by DC3, were measured in the SF of patients with OA. Finally, DC3s-CD8^+^ T cells formed a tight structure named ELLS. Similar lymphoid-like structures, such as tertiary lymphoid organs in tumors, have been previously noted [34, 35]. These structures are composed of various immune cell types, including dendritic cells and antigen-specific B and T lymphocytes. In contrast to secondary lymphoid organs, TLSs are not imprinted during embryogenesis, but are formed in nonlymphoid tissues in response to local inflammation. Before surgery, all enrolled patients with OA experienced pain with severe joint deformation. This may be related to the increased infiltration of DC3s into the synovium, which would prime more CD8^+^ T cells, causing more serious damage to the joint. Indeed, increased levels of TNF-*α* and IL12p70, of which DC3s are a major source, were observed in the SF of KL4 patients compared to KL3 patients. Although whether DC3s could predict poor OA prognosis needs to be further studied, our results strongly suggest that DC3s could play a critical role in OA pathogenesis and that targeting DC3s might be a novel therapeutic target to treat OA in the late phases.

## Data Availability

The datasets generated during and/or analyzed during the current study are available from the corresponding author on reasonable request.

## Supplementary Materials

Supplementary Materials Figure S1: gating strategy used to define immune cell types by flow cytometry. Figure S2: (A) proportions of each immune cell subset to CD45^+^ cells in PBMC, synovium, and SF. Red line presents 10% CD45^+^. (B) Counts of each immune cell subset *t* in PBMC, synovium, and SF. Figure S3: (A) distance analysis of DC3s and CD8^+^ T cell. Cellular phenotype of the fluorescence image depicted the spatial location of CD1c^+^CD163^+^ (red dots), CD163^+^ (green dots), and CD8^+^ (blue dots) in the synovium. Solid plots connected the nearest cells within 20 *μ*m from the CD1c^+^CD163^+^ and CD163^+^ to CD8^+^, respectively. (B) Association of DC3 infiltration with CD8^+^ T cells. Increased number of DC3 was measured when CD8^+^ T cells are located close to DC3s (<20 *μ*m). Each dot represents a single data point; blue dots represent ELLS-negative samples including OA37 and OA40, and violet dots represent ELLS-positive ones.
