# Supplementary material for: Expanded CD1c+CD163+ DC3 Population in Synovial Tissues Is Associated with Disease Progression of Osteoarthritis
Source: J Immunol Res. 2022 Aug 1;2022:9634073. doi: 10.1155/2022/9634073 (PMC9359855; doi:10.1155/2022/9634073)

Figure S1. Gating strategy used to define immune cell types by flow cytometry.

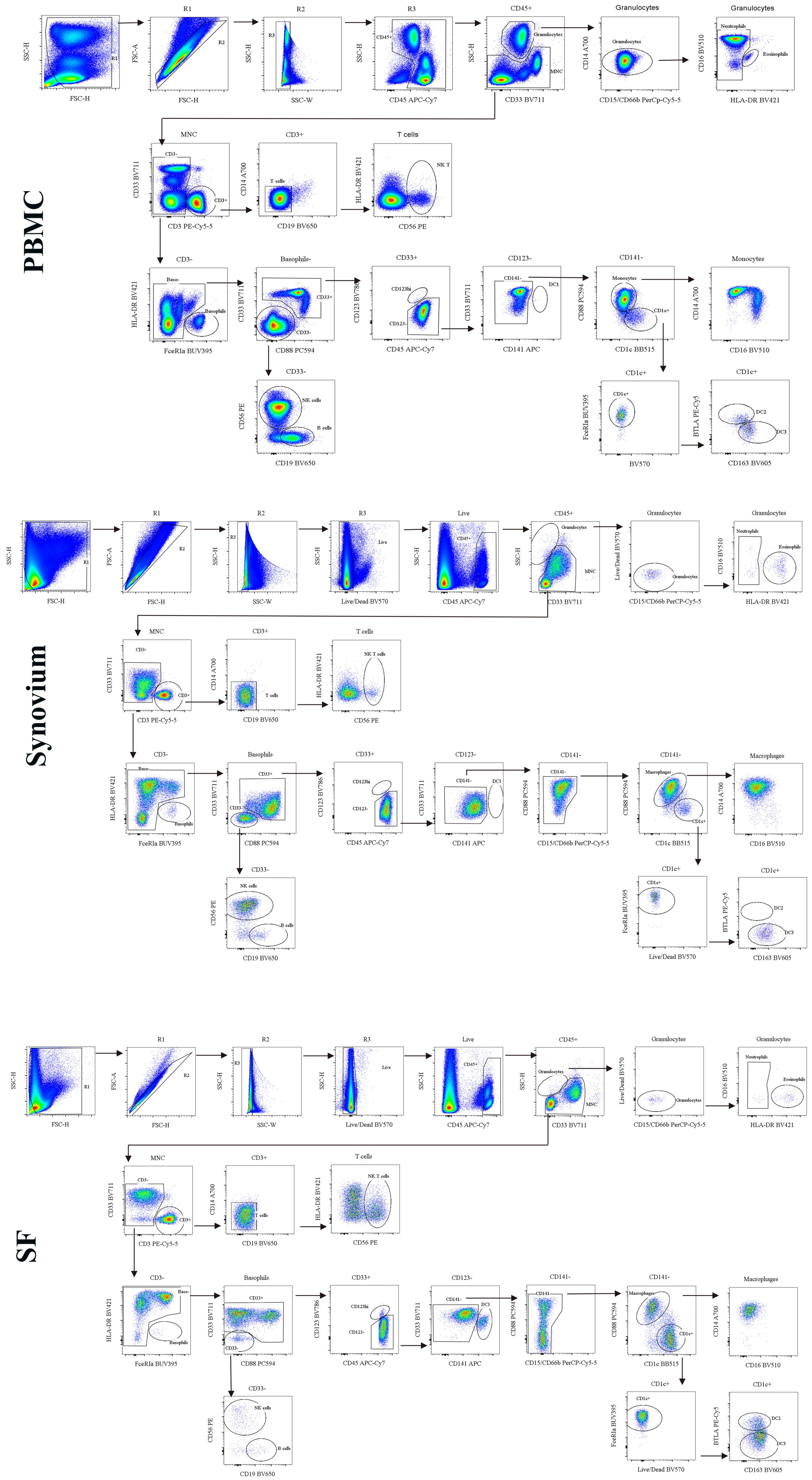

Figure S2. A. Proportions of each immune cell subsets to CD45+ cells in PBMC, synovium and SF. Red line present 10%CD45+.
B. Counts of each immune cell subsets t in PBMC, synovium and SF.

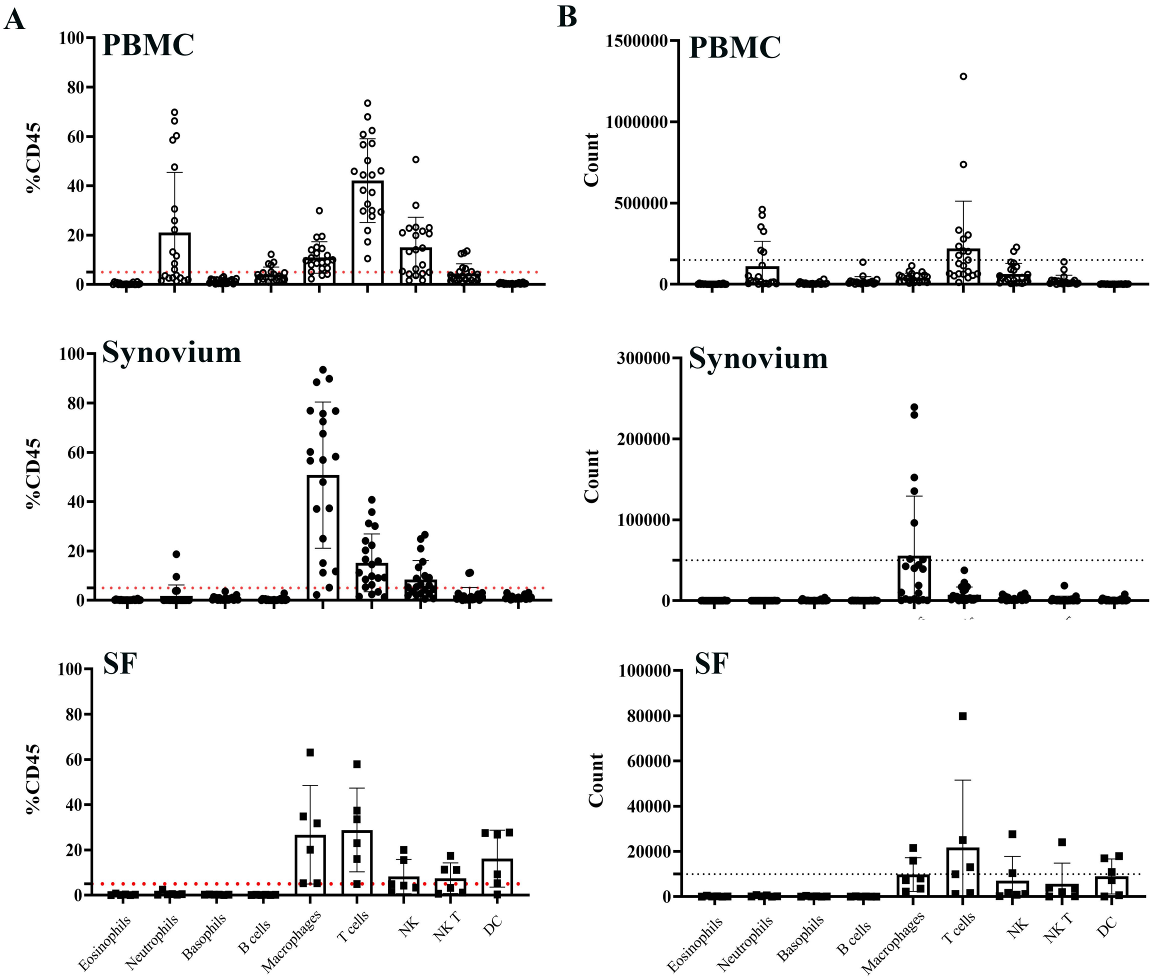

Figure S3. A. Distance analysis of DC3s and CD8+T cell. Cellular phenotype of the fluorescence image depicted the spatial location of CD1c+CD163+ (red dots), CD163 +(green dots) and CD8+(blue dots) in the synovium. Solid plots connected the nearest cells within 20 μm from the CD1c+CD163+ and CD163+ to CD8+ respectively.
B. Association of DC3s infiltration with CD8+ T cells. Increased number of DC3 were measure when CD8+T cells located close to DC3s (<20um). Each dot represents a single data point; blue dots represent ELLS negative samples including OA37 and OA40, and violet dots represent ELLS positive ones.

A

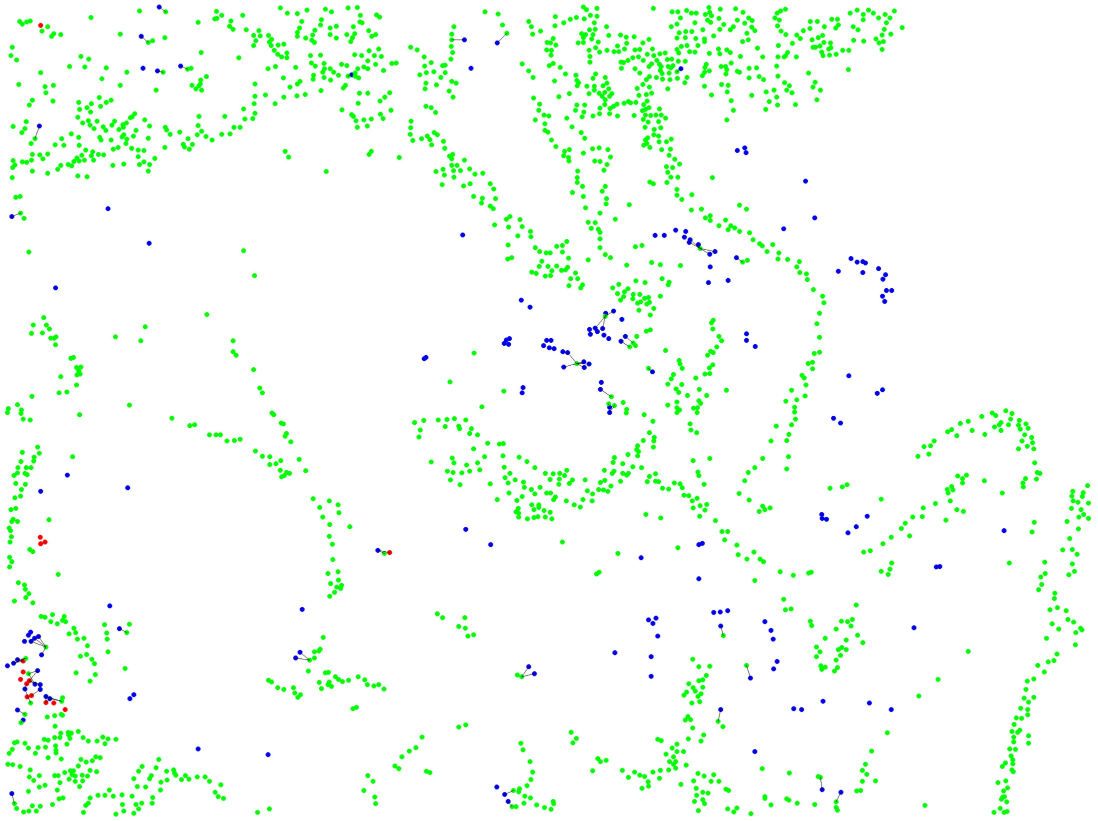

B

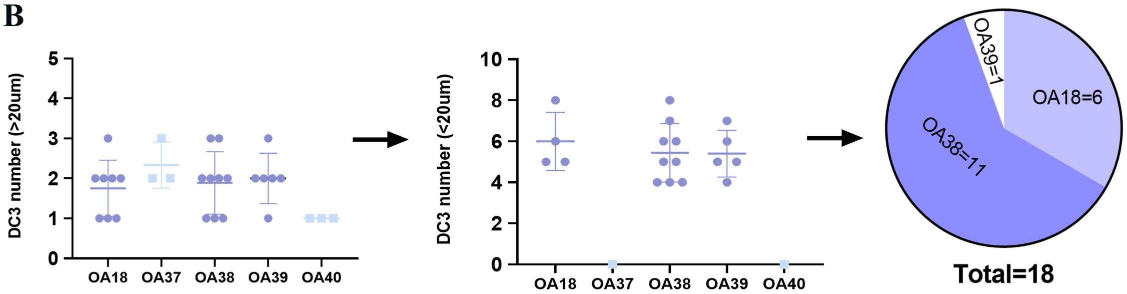

Supplement: Supplementary Materials — Figure S1: gating strategy used to define immune cell types by flow cytometry. Figure S2: (A) proportions of each immune cell subset to CD45+ cells in PBMC, synovium, and SF. Red line presents 10% CD45+. (B) Counts of each immune cell subset t in PBMC, synovium, and SF. Figure S3: (A) distance analysis of DC3s and CD8+ T cell. Cellular phenotype of the fluorescence image depicted the spatial location of CD1c+CD163+ (red dots), CD163+ (green dots), and CD8+ (blue dots) in the synovium. Solid plots connected the nearest cells within 20 μm from the CD1c+CD163+ and CD163+ to CD8+, respectively. (B) Association of DC3 infiltration with CD8+ T cells. Increased number of DC3 was measured when CD8+ T cells are located close to DC3s (<20 μm). Each dot represents a single data point; blue dots represent ELLS-negative samples including OA37 and OA40, and violet dots represent ELLS-positive ones. [file 9634073.f1.pdf]
